# Systems-Level Proteomic and Biochemical Profiling of Plasma from Captive Indian Star Tortoise with Reactome Pathway Enrichment Analysis

**DOI:** 10.3390/metabo16060398

**Published:** 2026-06-08

**Authors:** Dražen Đuričić, Josip Miljković, Krešimir Severin, Dominik Prišćan, Iva Šmit

**Affiliations:** 1 Department of Physiology and Radiobiology, Faculty of Veterinary Medicine, University of Zagreb, Heinzelova 55, 10000 Zagreb, Croatia; jmiljkovic@vef.unizg.hr; 2 Department of Pathobiology, Pharmacology and Zoological Medicine, Faculty of Veterinary Medicine, Ghent University, B-9820 Merelbeke, Belgium; 3 Department of Forensic and State Veterinary Medicine, Faculty of Veterinary Medicine, University of Zagreb, Heinzelova 55, 10000 Zagreb, Croatia; severin@vef.hr; 4 Internal Clinic, Faculty of Veterinary Medicine, University of Zagreb, Heinzelova 55, 10000 Zagreb, Croatia; dpriscan@vef.hr (D.P.); ismit@vef.unizg.hr (I.Š.)

**Keywords:** system-level proteomics, biochemical profile, *Geochelone elegans*, Reactome pathway

## Abstract

**Highlights:**

Findings reveal potential plasma biomarkers for health monitoring and diagnostics in In-dian star tortoises.
Identified 12 differentially expressed plasma proteins (nine upregulated, three downregulated).
Baseline plasma biochemical parameters to support future research.
Enriched pathways involved cytoskeletal organization, coagulation, and lipid metabo-lism.

**Abstract:**

**Background/Objectives:** The Indian star tortoise (*Geochelone elegans*) is a protected species for which physiological and molecular health indicators remain poorly characterized. This study aimed to monitor and analyze plasma proteome profiles and biochemical parameters in captive adult Indian star tortoises and to identify potential diagnostic biomarkers. **Methods:** Plasma samples from nine clinically healthy adult Indian star tortoises (four males and five females) maintained in captivity were subjected to biochemical profiling and proteomic analysis. Sex-related differences in biochemical parameters were evaluated, and differentially expressed proteins were mapped to *Homo sapiens* Reactome pathways to identify significantly enriched biological processes. **Results:** Plasma biochemical profiling established baseline reference values, indicating stable hepatic and metabolic function in captive tortoises. Creatinine and urea concentrations were significantly higher in females than in males (*p* < 0.05), suggesting sex-related differences in protein metabolism or renal function. No significant sex-related differences were observed in hepatic enzymes (ALP, ALT, AST, and GGT), muscle-associated enzymes (CK and LDH), glucose, cholesterol, triglycerides, total proteins, albumin, or electrolyte concentrations (Na, K, Ca, Mg, Cl, P, and Fe). Proteomic analysis identified 12 differentially expressed proteins, including nine upregulated and three downregulated proteins. Functional pathway analysis revealed 90 significantly enriched Reactome pathways (FDR < 0.05). Upregulated proteins were primarily associated with cytoskeletal organization (KRT75, KRT5, and KRT17), lipid transport and remodeling (APOB), coagulation (F10), extracellular transport (TTR), immune response (WFDC3), transmembrane signaling (KCP), and gamete interaction (ZAN). Downregulated proteins (C7, SERPING1, and PZP) were linked to complement activation and acute-phase response pathways. **Conclusions:** Captive Indian star tortoises exhibited increased cytoskeletal remodeling and coagulation activity together with reduced complement activation. These findings provide novel insights into the plasma proteome of this species and identify candidate biomarkers that may support future health assessment, physiological monitoring, and diagnostic applications in Indian star tortoises.

## 1. Introduction

The Indian star tortoise (*Geochelone elegans*, Schoepff, 1795) is a medium-sized terrestrial member of the family Testudinidae distributed across northwestern and peninsular India, Sri Lanka, and parts of Pakistan [1,2,3,4]. The species primarily inhabits dry forests, grasslands, and shrublands, but it is also frequently found in agricultural landscapes [4,5]. Although it is generally associated with arid environments, certain populations occur in more humid regions as well [6]. Recent observations indicate that individuals may swim and wallow during the monsoon season, suggesting behavioral flexibility and the possible use of hydrologically connected habitats within fragmented landscapes [4,7]. The species exhibits pronounced sexual dimorphism, with females generally exceeding 30 cm in length and males rarely surpassing 25 cm; males possess a concave plastron and longer tail, whereas females have a flat plastron and broader carapace [8,9]. They reach sexual maturity at approximately 6–8 years in males and 8–12 years in females [10]. Primarily herbivorous, the species feeds mainly on grasses and succulent plants but may opportunistically consume invertebrates [8,11]. Reproduction typically involves clutches of 3–7 eggs, with incubation periods ranging from 47 to 180 days [10,12]. Wild populations have declined significantly due to habitat loss, illegal collection, and wildlife trafficking; notably, more than 55,000 individuals were reported to have been exported from a single trade hub in India [1,13,14]. Consequently, the species is listed in the Convention on International Trade in Endangered Species of Wild Fauna and Flora (CITES, 2019) and is classified as Vulnerable on the International Union for Conservation of Nature Red List [2,9].

The chemical composition of blood serum or plasma represents an important indicator of individual fitness, health status, and physiology [15,16]. In chelonians, blood sampling and analysis of plasma biochemical parameters have become routine methodologies in veterinary practice and ecological research over the last decade, providing valuable insight into nutritional status, stress response, and disease condition [17,18]. However, despite an increasing body of literature on blood biochemistry in turtles and tortoises, comprehensive studies focused on the Indian star tortoise *G. elegans* remain conspicuously absent [1,2,9,19]. Species-specific physiological demands underscore the need for reliable reference intervals for blood biochemical parameters and for identifying sensitive biomarkers of health and metabolic status. Modern high-throughput proteomic technologies enable the identification, characterization, and quantification of a wide array of proteins in plasma and other body fluids, offering a powerful complement to classical biochemical analyses. Proteomic profiling has been used successfully in vertebrates to detect circulating biomarkers of environmental stress, disease states, and physiological imbalance, including tumors, cardiovascular conditions, and immune dysregulation [20,21,22]. In reptiles, omics-based studies addressing metabolic processes and protein modulation are still scarce, and plasma proteome analyses are notably underrepresented, with only a few reports focusing on soft tissues or specialized fluids in turtle species [22]. From a clinical and conservation perspective, the identification of reliable plasma biomarkers in *G. elegans* could enable non-invasive monitoring of health status, early detection of physiological disturbances, and evaluation of responses to rehabilitation or environmental stressors [23]. Combined biochemical and proteomic assessments offer complementary insights that can improve veterinary diagnostics, inform management decisions in captive care, and enhance monitoring of wild populations [24].

Despite its adaptability in the wild, captivity poses significant challenges. Inadequate husbandry is associated with high prevalence of endoparasites (i.e., cryptosporidiosis, etc.) and salmonellosis, highlighting the importance of biosecurity measures and regular health monitoring [25,26,27,28]. Conservation of *G. elegans* requires habitat protection, regulation of trade, and improvement in captive management. Molecular approaches, including proteomics and metabolomics, allow precise assessment of physiological and metabolic states, enable tracking of individual origins, and support successful reintroduction programs [2,5,13]. Here, for the first time, blood biochemical parameters and plasma proteome profiles were determined in adult Indian star tortoises in captivity. The study was designed to characterize baseline biochemical and proteomic profiles across age groups, to assess variations associated with physiological and environmental conditions, and to identify potential candidate biomarkers indicative of health status, metabolic balance, and adaptive responses, with implications for conservation and clinical care of this vulnerable species. Proteomic analysis, combined with pathway enrichment using Reactome, facilitates the identification of conserved molecular pathways involved in cytoskeletal organization, metabolism, coagulation, and immune regulation, providing mechanistic insights into health, adaptive responses, and metabolic status [29].

Despite the ecological and conservation importance of the Indian star tortoise, comprehensive integrative studies combining plasma biochemistry and proteomic profiling for health assessment and biomarker discovery in this species are currently lacking.

The study aims to investigate the proteomic profile and key biochemical parameters of the Indian star tortoise, integrating quantitative protein data with Reactome pathway analysis to identify molecular pathways and metabolic markers related to physiological function, health, and adaptive capacity, thereby informing conservation and captive management strategies.

## 2. Materials and Methods

The study was designed to characterize plasma biochemical parameters and proteome profiles in adult Indian star tortoises under captivity. The main experimental stages included the following: animal housing and biometric assessment, blood sampling, biochemical analysis, proteomics, pathway analysis and statistical analysis.

Controls: Biochemical assays included standard reference controls and triplicate measurements to ensure reproducibility. Proteomics analyses incorporated blank runs and technical replicates to monitor instrument performance and reduce false positives.

Limitations: Due to the absence of a fully annotated *G. elegans* proteome, human orthologs were used for pathway mapping, which may overlook species-specific proteins or post-translational modifications. Single-time-point blood sampling limits temporal resolution, and a small sample size may reduce statistical power for rare protein detection. The protocol is expected to be reliable for captive tortoises and for identifying conserved metabolic and immune pathways, but caution should be applied when extrapolating to wild populations or to species with substantially different physiology.

### 2.1. Materials

All chemicals were of analytical grade and complied with ACS specifications (Committee on Analytical Reagents of the American Chemical Society) or molecular biology grade standards. Bovine serum albumin (BSA, fraction V), acetone, acetonitrile, dithiothreitol (DTT), ethanol (96%), formic acid (96%), iodoacetamide (IAA), sodium acetate, sodium chloride, triethylammonium bicarbonate (TEAB), trypsin, and urea were purchased from Sigma-Aldrich (St. Louis, MO, United States of America (USA)). Calibration and quality control for biochemical analyses were performed using DiaSys TruCal U calibrator and TruLab N and TruLab P control sera. Ultrapure water (18.2 MΩ·cm resistivity, total organic carbon < 5 ppb) was generated using a Milli-Q Reference Water Purification System.

### 2.2. Animals

Nine Indian star tortoises (*G. elegans*) (four males and five females) confiscated from wildlife smugglers at a harbor on the Adriatic coast were included in this study. All animals were housed at the Faculty of Veterinary Medicine, University of Zagreb, Croatia, in individual enclosures and provided with a species-appropriate diet, heating lamps, and UVB lighting to ensure optimal husbandry conditions. No evidence was found to suggest that the environmental conditions, although considered optimal for the species, directly influenced the metabolic processes of the tortoises examined in this study. Blood samples were collected once from each individual during captivity. All procedures were conducted in compliance with national legislation, the EU Directive 2010/63/EU on the protection of animals used for scientific purposes, the ARRIVE guidelines, and the Croatian Animal Protection Act. The study protocol was approved by the Ethics Committee of the University of Zagreb Faculty of Veterinary Medicine (Class: 640-01/24-02/06; Reg. No.: 251-61-01/139-24-25; approval date: 23 May 2024). Morphometric measurements were recorded for each animal, including body mass (BM), straight carapace length (SCL), curved carapace length (CCL), carapace height (CH), carapace width (CW), plastron length (SPL), and anal plastron width (AFW). Body condition scoring was performed according to Lamberski (2011) [30]. Age classification was inferred from morphometric values, and all individuals were categorized as adults.

### 2.3. Blood Sampling

Blood sampling was performed approximately one year after the tortoises’ arrival at the facility. Up to two milliliters of blood were collected from the tail vein using a G23 needle attached to a syringe and transferred into BD Microtainer^®^ tubes containing lithium heparin (BD Diagnostics, NJ, USA). Tubes were gently inverted and incubated at room temperature (20–25 °C) for 15 min before centrifugation at 6800× *g* for 10 min at room temperature. The plasma supernatant was carefully harvested and stored at −80 °C until subsequent biochemical and proteomic analyses.

### 2.4. Biochemical Analysis

Plasma biochemical parameters were measured following standard protocols. Total protein, albumin, glucose, cholesterol, triglycerides, creatinine, bilirubin, urea, and other relevant analytes (ALP, ALT, AST, GGT, CK, LDH, amylase, lipase, lipase (DGGR), Mg, K, Na, Cl, Ca, P, and Fe) were determined at the Internal Clinic Laboratory of the University of Zagreb Faculty of Veterinary Medicine using an automated biochemistry analyzer (e.g., Olympus AU400/AU480, Beckman Coulter, Brea, CA, USA) with validated enzymatic and colorimetric assays suitable for reptilian plasma. Electrolytes (Na, K, and Cl) were measured using an ion-selective electrode (ISE) system integrated into the analyzer.

All measurements were performed in triplicate, and quality control samples were included to ensure accuracy and reproducibility. Data were used to evaluate baseline physiological status and to correlate biochemical markers with proteomic findings.

### 2.5. Reactome Pathway Enrichment Analysis

Pathway enrichment analysis was performed using the Reactome database (version 94, accessed on 22 September 2025), a curated resource of human biological pathways and reactions [31,32]. Reactome defines biological reactions as events that alter the state of molecules, including binding, activation, translocation, degradation, and enzymatic processes. The database content is manually curated and cross-referenced with multiple external resources such as NCBI, UniProt, KEGG, ChEBI, PubMed, and Gene Ontology (GO). For this analysis, differentially expressed proteins identified in the Indian star tortoise proteome were mapped to their human orthologs to leverage Reactome’s human pathway annotations [29]. An overrepresentation analysis (ORA) based on the hypergeometric distribution was conducted to identify pathways significantly enriched in the dataset.

### 2.6. Statistical Analysys

Statistical analyses were performed using R software version 4.4.1 (R Foundation for Statistical Computing, Vienna, Austria) with approaches appropriate for low-powered datasets. Descriptive data are presented as mean ± standard error of the mean (S.E.M.). Sex-related differences in biochemical parameters were assessed using the Mann–Whitney U test. Effect sizes (Cliff’s delta) and bootstrap 95% confidence intervals were calculated to provide magnitude estimates independent of *p*-values. Exact two-tailed *p*-values were reported, and *p* < 0.05 was considered statistically significant. Given the limited sample size, findings are interpreted as exploratory. Proteomic data were log2-transformed and analyzed using linear modeling with empirical Bayes moderation implemented in the limma package. Resulting *p*-values were adjusted for multiple testing using the Benjamini–Hochberg false discovery rate (FDR) correction. Proteins with FDR < 0.05 were considered significantly differentially expressed. Functional enrichment analysis was conducted using Reactome pathway annotations mapped to Homo sapiens due to the absence of species-specific databases for the Indian star tortoise.

### 2.7. Procedure

#### 2.7.1. Sample Preparation and Protein Digestion (Time: ~24 h)

Dilute 35 μg of plasma proteins in 100 mM triethylammonium bicarbonate (TEAB) buffer (pH 8.5). Add 2.5 μL of 200 mM dithiothreitol (DTT) and incubate the samples at 55 °C for 60 min to reduce disulfide bonds. Subsequently, add 2.5 μL of 375 mM iodoacetamide (IAA) and incubate for 30 min at room temperature (20–25 °C) in the dark to alkylate cysteine residues. Add 300 μL of ice-cold acetone and incubate samples overnight at −20 °C to precipitate proteins. Centrifuge at 8000× *g* for 10 min at 4 °C, discard the supernatant, and air-dry the pellet for 5 min. Resuspend the pellet in 50 μL of 50 mM TEAB buffer (pH 8.5). Add 1 μL of trypsin solution (1 mg/mL; enzyme-to-protein ratio 1:35) and incubate at 37 °C for enzymatic digestion.

CRITICAL STEP Perform alkylation in the absence of light to prevent degradation of IAA and ensure efficient cysteine modification.

CRITICAL STEP Avoid overdrying the protein pellet, as this may reduce solubility and digestion efficiency.

PAUSE STEP After digestion, samples can be stored at −20 °C for several days before further processing.

#### 2.7.2. Tandem Mass Tag (TMT) Peptide Labeling (Time: ~2–3 h)

Perform peptide labeling using the TMTsixplex Isobaric Label Reagent Set according to the manufacturer’s instructions. Add 19 μL of freshly prepared TMT labeling reagent (TMT6–127, TMT6–128, TMT6–129, TMT6–130, and TMT6–131) to each sample and incubate at room temperature for 60 min. Prepare an internal standard by pooling equal protein amounts from all plasma samples and label it with TMT6–126. Quench the labeling reaction by adding 8 μL of 5% hydroxylamine and incubating for 15 min at room temperature. Divide the labeled samples into nine groups by combining five randomly selected samples with the corresponding internal standard. Aliquot, dry, and store samples at −80 °C until LC–MS/MS analysis.

CRITICAL STEP Ensure equal protein input across samples to enable accurate relative quantification.

OPTIONAL STEP Verify labeling efficiency prior to LC–MS/MS analysis to ensure completeness of the reaction.

PAUSE STEP Labeled and dried samples can be stored at −80 °C for extended periods without degradation.

#### 2.7.3. LC–MS/MS Analysis (Time: ~24–48 h)

Perform high-resolution LC–MS/MS analysis using an UltiMate 3000 RSLCnano system coupled to a Q Exactive Plus mass spectrometer. Desalt peptides on a trap column (C18 PepMap100, 300 μm × 5 mm) and separate them on an analytical column (Pep-Map RSLC C18, 50 cm × 75 μm). Apply a linear gradient of 5–45% mobile phase B (0.1% formic acid in 80% acetonitrile) over 120 min at a flow rate of 300 nL/min. Use 0.1% formic acid in water as mobile phase A. Perform ionization using a nanospray ion source equipped with a 10 μm inner diameter emitter. Acquire full MS spectra in the m/z range of 350–1800 at a resolution of 70,000, with an AGC target of 1 × 10^6^ and an injection time of 120 ms. Select precursor ions using a data-dependent acquisition (DDA Top8) method with a ±2.0 Da isolation window and 30 s dynamic exclusion. Perform higher-energy collisional dissociation (HCD) fragmentation using stepped collision energy (29% and 35% NCE) at a resolution of 17,500 and AGC target of 2 × 10^5^. Exclude precursor ions with charge states of +1, >+7, or unassigned charge states.

CRITICAL STEP Maintain stable chromatographic conditions and mass accuracy to ensure reproducible peptide identification.

#### 2.7.4. Protein Identification and Quantification (Time: ~4–6 h)

Process raw data using the SEQUEST algorithm implemented in Proteome Discoverer (version 2.3). Perform database searches against reptilian protein sequences obtained from the National Center for Biotechnology Information. Set search parameters to allow up to two missed trypsin cleavages, with precursor and fragment mass tolerances of 10 ppm and 0.02 Da, respectively. Define carbamidomethylation (C) as a fixed modification and oxidation (M) and TMTsixplex labeling (K, peptide N-terminus) as dynamic modifications. Estimate the false discovery rate (FDR) using the Percolator algorithm and set it to 1% at the peptide level. Report only proteins identified with at least two peptides (including one unique peptide).

CRITICAL STEP Apply stringent FDR thresholds to ensure high-confidence protein identification.

#### 2.7.5. Reactome Pathway Enrichment Analysis (Time: ~1–2 h)

Upload the list of identified proteins (mapped to human orthologs) into the Reactome Pathway Browser. Perform pathway enrichment analysis using default statistical parameters and apply FDR correction to identify significantly enriched pathways.

CRITICAL STEP Interpret enriched pathways cautiously, considering cross-species orthology limitations.

OPTIONAL STEP Export pathway visualizations and enrichment tables for downstream analysis.

PAUSE STEP Analysis outputs can be stored and revisited without data loss.

#### 2.7.6. Biochemical Plasma Analysis (Time: ~2–4 h)

Perform biochemical analyses using automated clinical chemistry analyzers calibrated with DiaSys TruCal U and validated using TruLab N and TruLab P controls. Measure enzymatic and metabolic parameters, including ALP, ALT, AST, CK, LDH, glucose, total protein, and electrolytes.

CRITICAL STEP Perform calibration and quality control procedures prior to sample analysis to ensure accuracy and reproducibility.

Reagents and Materials

All chemicals were of high purity and ACS or molecular biology grade. Acetone, acetonitrile, bovine serum albumin (BSA), dithiothreitol (DTT), iodoacetamide (IAA), ethanol, formic acid, sodium acetate, sodium chloride, triethylammonium bicarbonate (TEAB), trypsin, and urea were obtained from Sigma-Aldrich. Calibration and control standards (DiaSys TruCal U, TruLab N, TruLab P) were used for biochemical analyses. Ultrapure water was prepared using the Milli-Q Reference Water Purification System.

## 3. Results

Of the submitted protein identifiers, eight out of 12 were successfully matched to Reactome entities, resulting in 90 pathway hits. The analysis was restricted to Homo sapiens pathways to ensure accurate functional annotation. Proteomic profiling identified 12 differentially expressed proteins (DEPs), including nine upregulated and three downregulated proteins (Table 1). Upregulated proteins were predominantly associated with cytoskeletal organization, coagulation, lipid metabolism, and innate immune responses. Notably, three keratin family members (KRT75, KRT5, and KRT17) involved in intermediate filament organization were increased. Proteins related to lipid transport and remodeling (APOB) and sperm–egg recognition (ZAN) were also elevated. Additionally, TTR, F10, WFDC3, and KCP were upregulated, indicating modulation of extracellular transport, coagulation pathways, antibacterial humoral response, and transmembrane transport processes. In contrast, the downregulated proteins were primarily linked to the complement system and acute-phase response. These included C7 and SERPING1, both involved in complement activation, as well as PZP, associated with acute-phase inflammatory processes.

**Figure 1:**
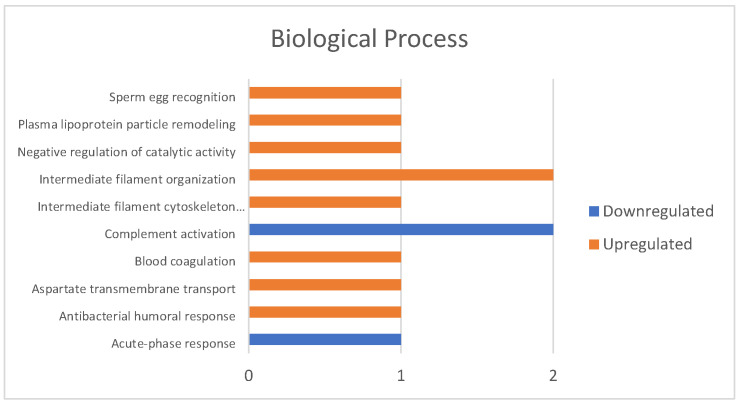
Distribution of upregulated and downregulated proteins according to Gene Ontology (GO) biological process categories in *G. elegans.*

**Figure 2:**
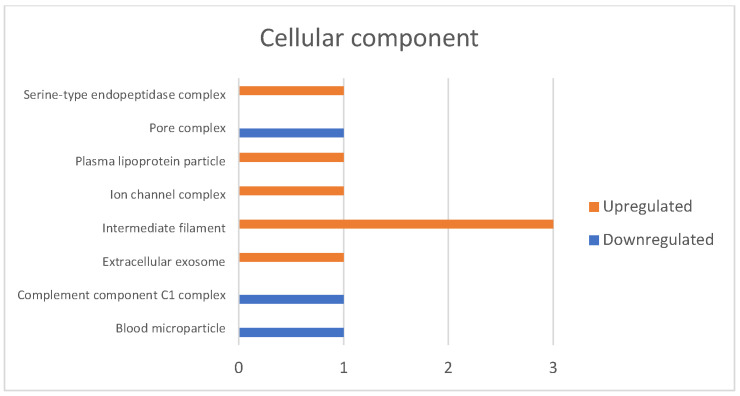
Classification of upregulated and downregulated proteins based on Gene Ontology (GO) cellular component annotation in *G. elegans.*

**Figure 3:**
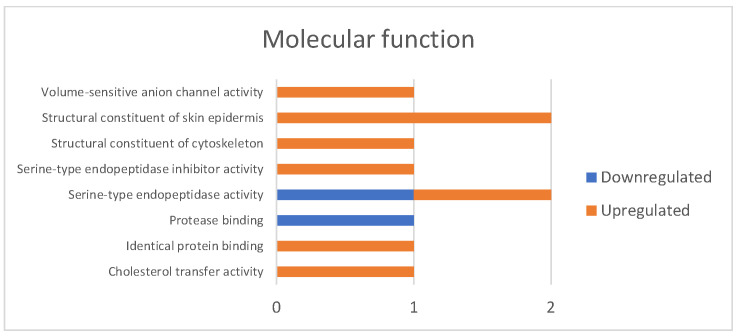
Distribution of upregulated and downregulated proteins based on Gene Ontology (GO) molecular function analysis in *G. elegans.*

Overall, the data suggest enhanced cytoskeletal remodeling and coagulation activity, accompanied by reduced complement activation under the studied condition. Gene Ontology (GO) classification of upregulated and downregulated proteins is presented in Figure 1 for biological processes, Figure 2 for cellular components, and Figure 3 for molecular functions.

**Figure 4:**
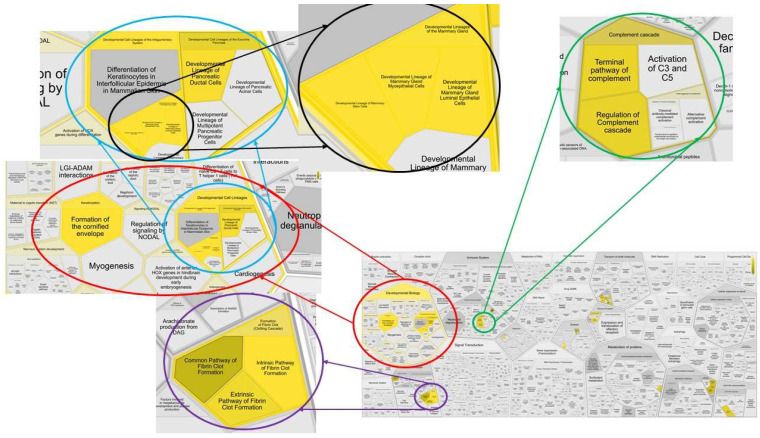
Functional classification of differentially expressed proteins in *G. elegans* using Reactome pathway analysis. Major functional clusters are highlighted and include the following: Developmental Biology (red cluster): Encompasses pathways related to cell lineage differentiation, such as the developmental lineage of multipotent pancreatic progenitor cells, mammary gland cells, and keratinocyte differentiation; Complement Cascade (green cluster): Includes the terminal pathway of complement activation, regulation of the complement cascade, and activation of complement components C3 and C5; Fibrin Clot Formation (purple cluster): Contains pathways involved in the common, intrinsic, and extrinsic pathways of fibrin clot formation; Developmental Lineages (blue cluster): Highlights specific developmental lineages such as mammary gland myoepithelial and luminal epithelial cells, as well as pancreatic ductal cells, indicating cellular differentiation dynamics relevant to tissue-specific functions.

Reactome pathway analysis of differentially expressed proteins in the Indian star tortoise revealed several major functional clusters (Figure 4). These clusters were primarily associated with developmental biology pathways related to cellular differentiation and lineage specification, complement cascade pathways involved in immune activation and regulation, fibrin clot formation pathways linked to coagulation processes, and developmental lineage pathways reflecting tissue-specific cellular differentiation dynamics.

Plasma biochemical analysis and proteomic profiling were performed on nine Indian star tortoises under captivity (Table 2). Urea concentrations were significantly higher in females compared to males (8.35 ± 2.8 vs. 3.2 ± 1.0 mmol/L; Mann–Whitney U = 1, *p* = 0.029). Creatinine concentrations were also higher in females (25.5 ± 2.0 vs. 19.5 ± 1.1 µmol/L), showing a strong trend toward significance (U = 2, *p* = 0.057). Effect size (Cliff’s delta) ≈ 0.75, indicating a large biological effect. No significant sex-related differences were observed in other biochemical parameters (*p* > 0.05), including hepatic enzymes (ALP, ALT, AST, GGT), muscle-associated enzymes (CK, LDH), metabolic indicators (glucose, cholesterol, triglycerides), total proteins, albumin, and electrolytes (Na, K, Ca, Mg, Cl, P, Fe).

Proteomic analysis of plasma using high-throughput mass spectrometry identified 20 significantly differentially expressed proteins, which were further mapped to human orthologs for Reactome pathway enrichment analysis. Key enriched pathways included intermediate filament organization (KRT17, KRT5, KRT75), blood coagulation cascade (F10, SERPING1), and plasma lipoprotein remodeling (TTR, APOB). All pathways were significant at FDR < 0.05, indicating robust enrichment (Table 3).

Morphometric characteristics of male and female *G. elegans* are summarized in Table 4. Females exhibited greater body mass and generally larger carapace and plastron dimensions compared to males, consistent with the pronounced sexual dimorphism reported for this species. Differences were particularly evident in curved carapace length, carapace height, carapace width, and plastron length. Body condition scores were comparable between sexes, indicating an overall good nutritional and physiological status of the examined individuals.

## 4. Discussion

The present study provides an integrated evaluation of morphometric characteristics, plasma biochemical parameters, and proteomic profiles in captive Indian star tortoises (*G. elegans*), offering novel insights into species-specific physiology and potential biomarker candidates. Functional interpretation of proteomic data was performed using the Reactome database through ortholog mapping. Although Reactome is human-curated, many fundamental biological processes—including cytoskeletal organization, coagulation cascades, complement activation, and lipid metabolism—are highly conserved across vertebrates. Therefore, homology-based pathway enrichment provides biologically meaningful insights while acknowledging potential species-specific regulatory differences. Consequently, identified pathways should be interpreted as conserved functional analogs rather than strictly human-specific mechanisms [31,32].

Keratin orthologs corresponding to human KRT5, KRT17, and KRT75 likely play conserved yet context-dependent roles in the integument of *G. elegans*. Keratin forms ~10 nm intermediate filaments composed of type I (acidic) and type II (basic/neutral) proteins that anchor to desmosomes, ensuring mechanical stability and epithelial integrity. In humans, keratin gene expression is highly differentiation-specific, with approximately half of the 54 keratin genes specialized for appendageal structures such as hair follicles [33,34,35,36]. In chelonians, these orthologs are likely adapted to support highly keratinized scute structures. Specifically, KRT17 is associated with activated keratinocytes and stress-responsive remodeling, suggesting a role in epidermal renewal and repair, whereas KRT75, a type II keratin, likely contributes to cytoskeletal resilience in basal epidermal layers. Together with KRT5, these proteins support a conserved intermediate filament network adapted to the mechanical demands of the chelonian shell and skin [33,34,35,36].

Although KRT5 and KRT17 are associated with basal epithelial identity in mammals, their presence in *G. elegans* does not imply conservation of mammary stem cell (MaSC) pathways. MaSCs originate from embryonic ectoderm and are regulated by WNT/β-catenin, FGF, NOTCH, Hedgehog, and steroid hormone signaling [37,38,39,40,41], and are primarily defined as bipotent epithelial progenitors in humans [42,43]. Given that reptiles lack mammary glands, keratin orthologs in *G. elegans* more plausibly reflect conserved structural roles in epithelial differentiation and the formation of a rigid protective integument [44,45,46,47].

Pathway enrichment analysis revealed significant overrepresentation of intermediate filament organization, coagulation pathways, and plasma lipoprotein remodeling following FDR correction [31,32]. The presence of keratin isoforms in plasma likely reflects physiological epithelial turnover or low-level tissue remodeling. Upregulation of coagulation factor X (F10) suggests modulation of hemostatic processes, potentially associated with tissue repair or stress responses. Concurrent enrichment of lipid transport proteins, including apolipoprotein B (APOB) and transthyretin (TTR), highlights metabolic adaptations relevant to reptilian physiology, particularly in relation to seasonal energy balance and reproduction [48]. Conversely, downregulation of complement-related proteins such as C7 and SERPING1 indicates modulation of innate immune pathways, which play a central role in reptilian immunity. Collectively, these findings suggest coordinated regulation of structural integrity, metabolism, coagulation, and immune function.

Despite reliance on human-based pathway annotation, orthology-driven inference remains a widely accepted approach for conserved biological systems. However, species-specific proteins and regulatory mechanisms may not be fully captured, and the results should therefore be considered hypothesis-generating. Enrichment of developmental biology pathways suggests ongoing tissue remodeling and maintenance processes, while activation of fibrin clot formation pathways supports a role for coagulation in vascular or repair mechanisms. Complement cascade modulation further reflects the dynamic regulation of inflammatory and immune responses.

Integration of biochemical and proteomic data revealed functional concordance between enzyme activity and protein expression. For instance, variation in plasma amylase and lipase corresponds with proteins involved in metabolic regulation and enzyme transport, while proteomically identified coagulation factors align with biochemical indicators such as creatine kinase (CK) and lactate dehydrogenase (LDH), reflecting systemic physiological homeostasis. Additionally, detection of intermediate filament proteins in plasma suggests their potential utility as sensitive biomarkers of tissue integrity and cellular stress.

Morphometric analysis confirmed pronounced sexual dimorphism, with females exhibiting greater body mass and larger carapace and plastron dimensions, consistent with reproductive and fecundity advantages [8,9,49,50,51]. Increased female body size is typically associated with enhanced energy storage and egg production capacity [6,52,53]. Comparable body condition scores between sexes indicate adequate husbandry conditions, minimizing confounding effects of malnutrition or chronic stress [54,55,56,57,58]. Nonetheless, sex-related differences in metabolic demands, particularly those linked to reproduction, may influence circulating biochemical and proteomic profiles.

Plasma biochemical parameters indicated overall stable hepatic, muscular, renal, and metabolic function, with most values falling within previously reported reference ranges. Enzymes such as ALP, ALT, AST, CK, and LDH, along with total proteins and electrolytes (Na, K, Ca), were consistent with physiological norms. Observed variability in CK, LDH, and amylase likely reflects individual differences, age, feeding competition, or handling-related stress [17,48,59]. Electrolyte balance remained stable, reflecting effective homeostatic regulation, which is particularly important in reptiles due to environmental influences on hydration and physiology [60]. Calcium and phosphorus dynamics are especially critical in chelonians, given their role in shell integrity and reproduction [61]. When compared with additional reference data (Exotic Animal Formulary), the majority of obtained values remained within expected ranges [62]. However, lipase activity was approximately six- to eight-fold higher than the ranges reported by Carpenter [62], and amylase values were also notably elevated. It should be noted that urea reference values are generally not reported in reptilian medicine, as uric acid is the primary parameter used for monitoring nitrogen metabolism and renal function. Furthermore, to the authors’ knowledge, this study presents, for the first time, reference values for magnesium (Mg) and iron (Fe) in this species, thereby contributing additional data to the existing biochemical baseline for reptiles.

The integration of omics and biochemical data strengthens the identification of non-invasive biomarkers for assessing physiological status in *G. elegans*. Such markers are particularly valuable in wildlife and conservation medicine, where minimally invasive approaches are essential [60,63]. Given the limited availability of reptilian proteomic data, this study contributes important baseline information for clinical assessment, rehabilitation, and conservation management. Considering the significant anthropogenic pressures on this species, including illegal trade and habitat fragmentation [1,2], the establishment of reliable physiological reference ranges and biomarker candidates has direct applications in health monitoring and conservation strategies.

## 5. Conclusions

In conclusion, this study provides a comprehensive physiological characterization of *Geochelone elegans* by integrating morphometric, biochemical, and proteomic analyses. The agreement between biochemical and proteomic findings highlights coordinated regulation of cytoskeletal organization, lipid metabolism, coagulation, and innate immunity. Identified proteins, particularly those related to keratin organization, lipid transport, and complement pathways, represent promising biomarkers for future validation and establishment of reference values in reptilian medicine. These findings provide valuable baseline data with potential applications in conservation medicine, captive management, and health monitoring of tortoise populations.

## Figures and Tables

**Table 1:** Differentially expressed proteins (DEPs) identified by proteomic analysis and their functional annotation based on Reactome pathway enrichment in captive *G. elegans.*

Accesion	Protein	Homolog	Gen
A0A8C0IXD7	Transthyretin	transthyretin precursor	TTR
A0A8C0INU5	Keratin 75	keratin, type II cytoskeletal 75	KRT75
A0A8C0H4F5	Keratin, type II cytoskeletal 5	keratin, type II cytoskeletal 5	KRT5
A0A8C0JD28	Phosvitin	zonadhesin isoform 6 precursor	ZAN
A0A8C0IX11	Keratin 17	keratin, type I cytoskeletal 17	KRT17
A0A452IUB9	Uncharacterized protein	kielin/chordin-like protein isoform 3 precursor	KCP
A0A452HDZ3	WAP four-disulfide core domain 3	WAP four-disulfide core domain protein 3 isoform	WFDC3
A0A8C0G226	Phosvitin	apolipoprotein B-100 precursor	APOB
A0A8C0IT40	coagulation factor Xa	coagulation factor X isoform 2	F10
A0A8C4WFE4	Complement component C7	complement component C7 precursor	C7
A0A8C0H9J3	Alpha-macroglobulin receptor-binding domain-containing protein	pregnancy zone protein precursor	PZP
A0A452HS35	Serpin domain-containing protein	plasma protease C1 inhibitor precursor	SERPING1

**Table 2:** Biochemical parameters of captive *G. elegans* (Mean ± S.E.M.).

Parameter	Overall (*N* = 9)	Males (*N* = 4)	Females (*N* = 5)	Unit
ALP	56.63 ± 11.78	63 ± 11.50	50.25 ± 8.88	IU/L
ALT	<0.4	<0.4	<0.4	IU/L
AST	42.00 ± 8.87	44.25 ± 5.25	39.00 ± 10.00	IU/L
GGT	2.13 ± 0.69	1.75 ± 0.75	2.50 ± 0.75	IU/L
CK	129.00 ± 90.75	180.00 ± 130.5 ^a^	78.00 ± 19.50 ^b^	IU/L
LDH	173.50 ± 44.13	189.25 ± 44.25	157.75 ± 37.75	IU/L
Amylase	1653.75 ± 545.75	1927.75 ± 443.25	1379.75 ± 409.63	IU/L
Lipase	38.50 ± 8.38	44.00 ± 13.50	33.00 ± 5.00	IU/L
Lipase (DGGR)	12.00 ± 1.33	12.50 ± 1.00	11.00 ± 2.00	IU/L
Glucose	3.18 ± 0.40	3.30 ± 0.50	3.05 ± 0.30	mmol/L
Cholesterol	2.68 ± 1.10	2.25 ± 0.78	3.10 ± 1.15	mmol/L
Creatinine	22.50 ± 3.25	19.50 ± 1.75 ^a^	25.50 ± 3.00 ^b^	μmol/L
Bilirubin	<0.9	<0.9	<0.9	μmol/L
Total Proteins	64.13 ± 8.59	66.25 ± 8.75	62.00 ± 9.50	g/L
Albumin	18.63 ± 2.78	19.00 ± 3.00	18.25 ± 2.75	g/L
Triglycerides	1.35 ± 1.09	1.65 ± 1.63	1.05 ± 0.70	mmol/L
Urea	5.78 ± 3.62	3.20 ± 1.40 ^a^	8.35 ± 4.55 ^b^	mmol/L
Mg	2.42 ± 0.39	2.45 ± 0.54	2.39 ± 0.25	mmol/L
K	5.98 ± 0.33	5.93 ± 0.18	6.03 ± 0.46	mmol/L
Na	128.00 ± 4.00	130.5 ± 4.00	125.5 ± 4.75	mmol/L
Cl	100.88 ± 3.13	101 ± 4.00	100.75 ± 2.25	mmol/L
Ca	3.61 ± 0.79	3.33 ± 0.51	3.83 ± 0.93	mmol/L
P	1.26 ± 0.41	1.28 ± 0.53	1.25 ± 0.30	mmol/L
Fe	8.74 ± 2.09	8.13 ± 2.16	9.65 ± 1.85	μmol/L

^a,b^ Superscript letters indicate statistically significant differences between groups (*p* < 0.05).

**Table 3:** Significantly enriched proteins and their associated genes in *G. elegans* plasma based on Reactome pathway analysis.

Associated Gene Names	*p*-Value	FDR *
KRT17, KRT5, KRT75	9.94 × 10^−11^	9.05 × 10^−9^
KRT17, KRT5, KRT75	2.98 × 10^−9^	1.34 × 10^−7^
KRT17, KRT5	4.20 × 10^−6^	1.26 × 10^−4^
KRT17, KRT5	8.17 × 10^−6^	1.80 × 10^−4^
KRT17, KRT5	2.35 × 10^−5^	4.24 × 10^−4^
KRT17, KRT5	9.46 × 10^−5^	1.35 × 10^−3^
KRT17, KRT5, KRT75	1.04 × 10^−4^	1.35 × 10^−3^
KRT17, KRT5	2.39 × 10^−4^	2.57 × 10^−3^
F10, SERPING1	2.56 × 10^−4^	2.57 × 10^−3^
KRT17, KRT5	3.10 × 10^−4^	2.68 × 10^−3^
F10, SERPING1	3.34 × 10^−4^	2.68 × 10^−3^
F10, SERPING1	3.92 × 10^−4^	2.74 × 10^−3^
F10, SERPING1	1.06 × 10^−3^	7.42 × 10^−3^
KRT17	3.32 × 10^−3^	1.75 × 10^−2^
SERPING1	3.35 × 10^−3^	1.75 × 10^−2^
TTR, APOB	3.49 × 10^−3^	1.75 × 10^−2^
TTR, APOB	4.41 × 10^−3^	1.78 × 10^−2^
F10	4.46 × 10^−3^	1.78 × 10^−2^
F10	4.46 × 10^−3^	1.78 × 10^−2^
TTR	4.46 × 10^−3^	1.78 × 10^−2^

* All listed pathways and associated genes have FDR < 0.05, indicating statistically significant enrichment.

**Table 4:** Morphometric characteristics of male (*N* = 4) and female (*N* = 5) *G. elegans* (mean ± S.E.M.).

Parameter	Unit	Males (Mean ± S.E.M.)	Females (Mean ± S.E.M.)
Body weight	g	1443.75 ± 116.63	2144.40 ± 434.18
SCL (Straight carapace length)	cm	24.08 ± 2.46	24.20 ± 1.16
CCL (Curved carapace length)	cm	28.63 ± 1.49	31.18 ± 2.06
CH (Carapace height)	cm	11.50 ± 0.60	13.04 ± 1.17
CW (Carapace width)	cm	13.45 ± 0.63	16.50 ± 1.36
SPL (Straight Plastron length)	cm	16.15 ± 2.43	20.40 ± 1.48
AFW (Anal plastron width)	cm	5.05 ± 0.25	5.30 ± 0.48
BCS (Body condition score)	–	5.08	5.56

## Data Availability

The datasets generated and/or analyzed during the current study are available in the Supplementary Materials accompanying this article.

## Supplementary Materials

The following supporting information can be downloaded at: https://www.mdpi.com/article/10.3390/metabo16060398/s1. Table S1: Significantly enriched Reactome pathways; Table S2: Significantly enriched pathways with associated genes, false discovery rate (FDR), and p-values; Table S3: Reactome pathway analysis of IZUMO1R; Table S4: Reactome pathway analysis of AHSG; Table S5: Molecular function enrichment analysis of AHSG; Table S6: Biological process enrichment analysis of AHSG; Table S7: Biological process enrichment analysis of IZUMO1R; Table S8: Reactome pathway analysis of LYG2; Table S9: Identified proteins, homologous proteins, and corresponding homolog genes; Table S10: Plasma biochemical parameters of the studied Indian star tortoises (*Geochelone elegans*).

## Abbreviations

The following abbreviations are used in this manuscript:

AFW	Anal Plastron Width
ALP	Alkaline phosphatase
ALT	Alanine aminotransferase
AST	Aspartate aminotransferase
APOB	Apolipoprotein B
BM	Body Mass
Ca	Calcium
C7	Complement component 7
Cl	Chloride
CCL	Curved Carapace Length
CH	Carapace Height
CK	Creatine kinase
CW	Carapace Width
F10	Coagulation factor X
FDR	False discovery rate
FGF	Fibroblast growth factor
GO	Gene Ontology
K	Potassium
KRT5	Keratin 5
KRT17	Keratin 17
KRT75	Keratin 75
LDH	Lactate dehydrogenase
MaSCs	Mammary stem cells
Na	Sodium
NOTCH	Notch signaling pathway
SCL	Straight Carapace Length
SERPING1	Serpin family G member 1
SPL	Straight Plastron Length
TTR	Transthyretin
WNT	Wingless-related integration site signaling pathway
ZAN	Zonadhesin isoform 6 precursor
